# An interprofessional approach to improving paediatric medication safety

**DOI:** 10.1186/1472-6920-10-19

**Published:** 2010-02-19

**Authors:** Moira Stewart, Joanna Purdy, Neil Kennedy, Anne Burns

**Affiliations:** 1 Department of Child Health, Queen's University Belfast, Grosvenor Road, BT12 6BP, Belfast, Northern Ireland; 2 Centre for Excellence in Interprofessional Education, School of Dentistry, Queen's University Belfast, Grosvenor Road, BT12 6BP, Belfast, Northern Ireland; 3 Royal Belfast Hospital for Sick Children, Grosvenor Road, BT12 6BP, Belfast, Northern Ireland

## Abstract

**Background:**

Safe drug prescribing and administration are essential elements within undergraduate healthcare curricula, but medication errors, especially in paediatric practice, continue to compromise patient safety. In this area of clinical care, collective responsibility, team working and communication between health professionals have been identified as key elements in safe clinical practice. To date, there is limited research evidence as to how best to deliver teaching and learning of these competencies to practitioners of the future.

**Methods:**

An interprofessional workshop to facilitate learning of knowledge, core competencies, communication and team working skills in paediatric drug prescribing and administration at undergraduate level was developed and evaluated. The practical, ward-based workshop was delivered to 4^th ^year medical and 3^rd ^year nursing students and evaluated using a pre and post workshop questionnaire with open-ended response questions.

**Results:**

Following the workshop, students reported an increase in their knowledge and awareness of paediatric medication safety and the causes of medication errors (p < 0.001), with the greatest increase noted among medical students. Highly significant changes in students' attitudes to shared learning were observed, indicating that safe medication practice is learnt more effectively with students from other healthcare disciplines. Qualitative data revealed that students' participation in the workshop improved communication and teamworking skills, and led to greater awareness of the role of other healthcare professionals.

**Conclusion:**

This study has helped bridge the knowledge-skills gap, demonstrating how an interprofessional approach to drug prescribing and administration has the potential to improve quality and safety within healthcare.

## Background

Safe drug prescribing and administration are essential elements within undergraduate healthcare curricula. However, a number of studies have reported that medical students feel unprepared for this aspect of clinical practice and on completion of undergraduate training they would not meet the competencies identified by the General Medical Council [1-3]. Nurses have reported limited understanding of pharmacology, dissatisfaction with the pre-registration teaching of the subject and feeling unprepared to perform certain tasks within nursing practice because of a lack of knowledge about the drugs they administer [4,5].

Drug prescribing is very different in adult and paediatric practice. Infants and children are prescribed fewer drugs than adults, but are at a disproportionately higher risk of medication errors [6]. Drug prescribing and administration is more complex in children: drug doses need to be calculated on an individual basis taking into account gestation and postnatal age, weight and/or body surface area [7,8]. Drugs prescribed for children are often unlicensed and formulated for adults [9].

Medication errors, particularly in paediatric practice, have been attributed to a lack of knowledge and skills [10,11] resulting from inadequate education and training [7,12]. Poor mathematical skills have been demonstrated among medical and nursing students, further increasing the risk of medication errors following qualification [13-15]. In addition, errors in drug prescribing and administration have been attributed to poor communication and lack of teamworking between healthcare professionals [16]. In paediatric practice, involvement of family members further complicates the prescribing process [17].

### Aim

The aim of this study was to develop and evaluate an interprofessional teaching and learning workshop of paediatric dug prescribing and administration for medical and nursing students, which would facilitate learning of knowledge, core competencies, communication and team working skills. In addition, rigorous evaluation of the workshop could inform curriculum development.

## Methods

### The Centre for Excellence in Interprofessional Education (CEIPE)

CEIPE was established to identify and develop opportunities within healthcare and other academic disciplines, to enhance students' team working and communication skills, promote collaborative practice and ultimately, improve patient care. In collaboration with the Schools of Medicine, Dentistry and Biomedical Sciences and Nursing and Midwifery at Queen's University Belfast, 'Paediatric Drug Prescribing and Administration' was identified as an appropriate area for teaching and learning in an interprofessional context for undergraduate medical and nursing students.

This work received approval from the Office for Research Ethics Committees Northern Ireland (05/NIR01/167) on 4 January 2006.

### Interprofessional Education Group

An interprofessional education (IPE) group comprising paediatricians, a paediatric clinical pharmacist, a nurse educator and research staff was established to develop and evaluate an interprofessional programme of teaching and learning to address medication safety issues in paediatric practice.

### Learning Outcomes

The IPE group identified common learning outcomes (Table 1) for the workshop with particular emphasis on practical, communication and team working skills.

**Table 1:** Student learning outcomes for Paediatric Drug Prescribing and Administration Workshop

The successful student should be aware of:
• the collective and individual responsibilities in drug prescribing and administration
• relevant sources of information on prescribing for children (British National Formulary for Children/Local Guidelines/Drug Information Services/Pharmacists)
• common sources of errors in paediatric drug prescribing (calculation errors/appropriate formulation/body weight calculations)
• practical issues in drug administration
• importance of information (written and verbal) and communication with other professionals and parents (indications for use/side effects/use of "off-label" medication)

The successful student should be:
• able to accurately complete a Drug Kardex

The successful student should:
• recognise that accurate drug prescribing and administration are essential skills in safe paediatric practice

### Students

Fourth year paediatric medical and 3^rd ^year children's branch nursing students had completed core teaching (see below) and were identified as the most suitable groups to participate in the workshop [18].

### Teaching programme

#### i. Core Teaching

During the 'Healthcare of Children' module, 4^th ^year medical students receive a lecture in 'Pharmacokinetics and Prescribing in Infants and Children' delivered by a paediatric clinical pharmacist. Information is provided on children's physiological response to drugs, prescribing and dosage regimes and common causes of errors in paediatric prescribing. In year 2 of their degree, nursing students also receive teaching by a pharmacist, which includes paediatric drug dosage, drug interactions, legal aspects and calculations. Nurse lecturers deliver opportunistic teaching on drugs used for common conditions and their side effects during year 3 of the children's nursing curriculum.

#### ii. Interprofessional Workshop

A practical, ward-based workshop, facilitated by the IPE group on paediatric drug prescribing and administration was delivered to five groups of medical and nursing students during 2007-2008. Four 'real-life' clinical scenarios (Table 2) were constructed and included medications commonly used in paediatric clinical practice. Students were allocated to small interprofessional groups (n = 2-3) and were required to prescribe the appropriate drug, calculate the correct dosage, accurately complete a Drug Kardex, prepare the drug for administration, identify alternative drugs where appropriate, prepare an intravenous infusion and to be able to provide information to parents. The tasks varied in complexity and in the skills required, but all were dependent on students having prior knowledge of basic pharmacology and also on communication and team working, with complementarity of the specific uniprofessional expertise.

**Table 2:** Examples of 'Real-Life' Clinical Scenarios

Summary of Clinical Scenario	Drug	Questions for students	Resources Required
18 month old admitted to hospital following simple febrile illness. Red ears and throat and probable viral upper respiratory tract infection.	Ibuprofen or Paracetamol	1. What is first line antipyretic agent? 2. Calculate dose, complete Drug Kardex and prepare for administration. 3. When nurses try to administer drug, child screams and spits it out immediately. What should be done next?	• BNFC • Medicine (syrup, suppository, tablet) • Drug Kardex • Discharge proforma • Oral syringe
7 year old with chronic asthma who is admitted with a severe acute attack. Child regularly uses inhalers and oral medication. Child responds poorly to treatment with oxygen, 'back-to-back' nebulisers and steroids.	Intravenous Aminophylline	1. What dose should be prescribed? 2. What other information is needed? 3. Write a script for intravenous aminophylline infusion. 4. What monitoring is required? 5. Make up an intravenous aminophylline infusion.	• Intravenous Aminophylline ampoules (2) • Large 20-30 ml syringe & needles • NaCl 0.9% 500 ml or 5% dextrose & fluid pump & associated giving set • Filter needle • 'Sharps box'

### Resources

Student and tutor study guides were developed which included the learning outcomes, guidelines for conducting the workshop, four clinical scenarios and resources required.

Students received a certificate from CEIPE acknowledging their attendance and participation in the workshop to be included in their portfolio.

### Research and Evaluation

The workshop was evaluated using pre and post-workshop questionnaires based on the previously validated 'Readiness for Interprofessional Learning Scale'[19,20]. The 19 statement questionnaire used a 5-point Likert scale, ranging from strongly disagree to strongly agree, and examined student attitudes to shared learning, development of communication and team working skills, knowledge and awareness of medication safety in children and awareness of professional roles and responsibilities. The questionnaire also included open-ended response questions, allowing students to comment on this particular interprofessional learning experience.

Pre-workshop questionnaires were completed by 4^th ^year medical students (n = 172) undertaking the Healthcare of Children Module. Post-workshop questionnaires were administered to the medical students (n = 48) who participated in the workshop during their paediatric clinical attachment in the Royal Belfast Hospital for Sick Children. Pre and post-workshop questionnaires were completed by 3^rd ^year nursing students (n = 21) who participated in the workshop (one post-workshop questionnaire missing).

### Data Analysis

Questionnaire data were analysed using SPSS version 15.0. The 5 point Likert scale was converted to 0-100. Factor analysis was applied to reduce the original 19 Likert scale questionnaire statements to a smaller number of agreed domains (Table 3). Chronbach's Alpha (α) measure of reliability was used to determine the internal consistency of each domain. Domains with a Cronbach's α > 0.69 were accepted as reliable and used in the data analysis [21]. A General Linear Model was used to compare mean pre and post workshop scores, taking account of group effects and group by time interactions. Further analysis used the same approach to compare mean pre and post workshop scores for each discipline.

**Table 3:** Questionnaire statements categorised into 4 domains

**Domain 1: Shared Learning**
1. Shared learning will increase my ability to understand drug prescribing and administration in paediatrics.
2. Drug prescribing and administration can only be learnt effectively with students from my own discipline.
3. Drug prescribing and administration can only be learnt effectively with students from a number of difference disciplines.
4. Shared learning will help me understand my own professional limitations.
**Domain 2: Skills Development**
5. Team working skills are essential for all healthcare students to learn and work together.
6. Patients would ultimately benefit if healthcare students worked together to ensure safety in drug prescribing and administration among children.
7. Shared learning will help me to communicate better with children, parents and other healthcare professionals.
8. I feel interprofessional communication is essential to ensure mistakes are avoided in the prescribing and administration of drugs.
**Domain 3: Knowledge and awareness**
9. I have little or no knowledge of the main sources of information on prescribing and administering drugs for children.
10. I am aware of the common sources of error in paediatric drug prescribing and administration.
11. I have a good understanding of the practical drug prescribing and administration issues which may arise with children.
12. I can accurately complete a Drug Kardex.
**Domain 4: Role Awareness**
13. I am aware of my role and responsibility in the safe prescribing and administration of drugs in paediatrics.
14. I am aware of the role and responsibility of other healthcare professionals in safe prescribing and administration of drugs in paediatrics.
15. I feel other healthcare professionals do not fully understand my role in prescribing or administering drugs to children.
16. I am anxious about approaching other healthcare professionals about a drug prescribing and administration safety issue.
17. I feel confident in approaching more senior members of staff about a drug prescribing and administration safety issue.
18. I am receptive to other healthcare professionals approaching me about drug prescribing and administration safety issue.
19. My clinical practice will change as a result of participating in interprofessional training in drug prescribing and administration for children.

The open-ended responses were thematically analysed using the principles of Grounded Theory [22]. The emergent themes were verified independently by two researchers [23,24].

## Results

The interprofessional workshop was conducted over five 2 hour sessions during the 2007-2008 academic year, with participation from 48 medical and 21 nursing students.

Analysis of the pre-workshop mean scores for the whole medical student group (n = 172) with those students who undertook their clinical attachment in the Royal Belfast Hospital for Sick Children revealed no significant differences between the groups. Therefore, it was deemed acceptable to use the pre-workshop responses for all 172 medical students. Nursing students (n = 21) completed pre and post workshop questionnaires (one post-workshop questionnaire was not completed).

The demographic profile of the workshop participants is shown in Table 4. Almost half of the nursing students had a previous undergraduate degree and approximately two thirds of students had previous experience of interprofessional teaching and learning activities. One third of medical students (n = 18) and almost all nursing students (n = 19) reported to have had previous teaching in drug prescribing and administration.

**Table 4:** Demographic profile of workshop participants

		Medical Students	Nursing Students	Total
**Gender**	Male	18	1	19
	Female	30	19	49
**Age**	18-22	33	8	41
	23-28	15	7	22
	29-34	0	2	2
	Over 34	0	3	3
**Previous Degree**	Yes	1	9	10
	No	47	11	58
**Previous shared learning experience**	Yes	27	16	43
	No	21	4	25
**Previous related teaching**	Yes	18	19	37
	No	30	1	31

Following data analysis, the 19 statement questionnaire was reduced to 3 domains (Table 5). One domain (Role Awareness) was excluded from further analysis due to a Cronbach's Alpha score <0.69, indicative of poor internal consistency between the statements within this particular domain. The results from Tables 5, 6 and 7 are reported collectively by domain.

**Table 5:** Pre and post questionnaire mean domain scores for paediatric drug prescribing and administration workshop

Domains	Mean Score		Mean Difference	95% Confidence Interval
	Pre	Post		
Knowledge and Awareness^a^	53.9	69.8	15.9	10.4 - 21.4***
Shared Learning	67.9	76.6	8.7	4.3 - 13.1***
Communication and Teamworking	81.4	82.5	1.1	-2.6 - 4.9

^a ^Adjusted for group effects and group by time interactions *** p < 0.001

**Table 6:** Pre and post workshop mean domain scores by discipline (medicine)

Domains	Nursing		Mean Difference	95% Confidence Interval
	Pre	Post		
Knowledge and Awareness^b^	43.0	65.9	22.9	17.8 - 28.0***
Shared Learning	67.4	76.7	9.3	4.0 - 14.6***
Communication and Teamworking	81.1	83.9	2.7	-1.8 - 7.3

^b ^Adjusted for effects of previous teaching in this subject area *** p < 0.001

**Table 7:** Pre and post workshop mean domain scores by discipline (nursing)

Domains	Nursing		Mean Difference	95% Confidence Interval
	Pre	Post		
Knowledge and Awareness^b^	65.2	72.2	7.0	-1.1 - 15.1
Shared Learning	72.6	75.8	3.2	-5.5 - 11.8
Communication and Teamworking	83.3	80.3	-3.0	-10.2 - 4.1

^b ^Adjusted for effects of previous teaching in this subject area

Pre workshop scores were high for all domains, indicating positive attitudes, particularly in the areas of shared learning and communication and teamworking. Mean post workshop scores (Table 5) indicate a positive shift in student attitudes to shared learning, communication and teamworking, and knowledge and awareness.

### Knowledge and Awareness

The greatest change in students' responses was observed within the 'Knowledge and Awareness' domain, where a highly significant arbitrary difference was observed in pre and post workshop scores. Students reported considerable improvement in their knowledge and awareness of paediatric medication safety and the causes of medication errors. In particular, highly significant differences were observed in the medical students' mean pre and post workshop scores. Although similar post workshop scores were observed for both student groups, the reported increase was significantly greater for medical students (pre 43.0; post 65.9, p < 0.001) compared with nursing students (pre 65.2; post 72.2).

### Shared Learning

Highly significant changes in students' attitudes to shared learning (pre 67.9; post 76.6; p < 0.001) were observed following the workshop. Students reported that paediatric drug prescribing and administration are learnt more effectively with students from other healthcare disciplines than in a uniprofessional context. Participation in the workshop also enabled students to recognise their own professional limitations.

When analysed for group effects, there was a highly significant positive shift in medical students' attitudes to shared learning following participation in the workshop. Nursing students were more positive than medical students prior to the workshop which may account for the smaller increase in post workshop scores.

### Communication and Teamworking

In the domain of Communication and Teamworking, no significant differences were observed in pre and post workshop scores, or within each discipline.

### Results from open-ended questions

Three key themes emerged from the data - value of the learning experience, relevance to future practice and learning challenges.

#### Theme: Value of the learning experience

Students found the interprofessional workshop to be worthwhile, useful informative and enjoyable and a valuable *practical *learning experience. Medical students in particular, reported that this was their first opportunity to practise prescribing skills.

#### Theme: Relevance to Future Practice

A small number of students reported that the 'hands-on' experience, coupled with their clinical placement, would be of benefit in their future practice.

Practical points cannot be learned effectively from textbook. Worthwhile exercise following through with scripts and preparation. (Medical Student)

Students reported the experience was relevant to future practice as it increased knowledge of drugs, administration and prescribing and particularly of drug calculations, medication doses and preparation and the use of relevant sources of information. Students reported increased confidence resulted and that this was important for future practice. As well as increased knowledge students reported the experience improved their practical skills in prescribing, communication and team working.

Nursing students particularly recognised the potential benefits of learning and working together as a team.

To be able to work together with doctors and learn from each other. (Nursing Student)

Additionally, students reported that the interprofessional learning experience was relevant to future practice as it increased their awareness of medication errors common in paediatric practice and the role of other healthcare professionals in the drug prescribing and administration process. It also enabled them generally, to learn more about the role of other healthcare professionals which was reported as being relevant to future work.

#### Theme: Learning Challenges

Some students reported difficulty with the mathematical calculations, whilst others felt that more time should be allocated to the workshop to allow for more detailed consideration and discussion of the clinical scenarios. Students also reported that they would prefer an equal number of students from each discipline (in the present study this was not possible, since there are fewer nursing than medical students) and that they would benefit from greater insight into the role of the other healthcare professionals. Students also highlighted practical issues such as individual access to a British National Formulary, larger teaching space and more students participating in the workshop.

## Discussion

The design and delivery of this interprofessional workshop resulted in quantitative and qualitative evidence of improvement in clinical knowledge and skills and a greater awareness of the importance of shared learning. In addition, the qualitative data supported the value medical and nursing students placed on communication and teamworking skills in relation to learning and working together. Students were positive about the practical nature of the workshop, recognising its application to clinical practice in bridging the knowledge-skills gap in both prescribing and administration of drugs and awareness of potential medication errors. Their responses support the key elements for successful educational interventions, which emphasise the importance of a strong practical approach to teaching [25].

We attempted to address two issues when designing this workshop. Firstly, concerns among newly qualified doctors and nurses about their preparedness for safe drug prescribing and administration. Even though all students, medical and nursing, had been timetabled to have teaching in pharmokinetics, specific to paediatric drug prescribing and administration, two-thirds of medical students reported that they had not received any such teaching. This raises concerns about students' perception of the importance and relevance of theoretical teaching and their uptake of classroom based lectures in the absence of opportunities for practical application.

Secondly, in light of evidence of the importance of communication between healthcare professionals for safe medication practice, communication and teamworking and appreciation of the skills and expertise of other professionals were integral learning outcomes for the workshop. Evidence to date highlights the importance of communication between healthcare professionals for safe medication practice [16]. Failure in any one of these competencies can lead to a cumulative series of errors involving various members of the healthcare team, with adverse patient outcomes. In one study it was reported that 81% of medication errors could have been avoided with pharmacist monitoring, 47% could have been avoided by improved communication between physicians and pharmacists and 17% could have been prevented with improved communication between doctors and nurses [16].

If we accept the need for improved teaching and training of knowledge and skills required for safe drug prescribing and administration, it seems evident that equal attention should be directed to communication and teamworking skills. According to the World Health Organisation [26] communication and teamworking skills, essential for collaborative practice, are best learned in an interprofessional context, but debate continues as to the optimal stage of the curriculum at which interprofessional learning should be introduced and the environment in which it should be taught [27]. In this study, where the emphasis was on practical rather than theoretical aspects of paediatric prescribing and administration, there was qualitative evidence of the development of communication and teamworking skills. An interprofessional approach, involving students from different professional backgrounds has the potential to meet these various learning objectives and improve clinical practice.

It was apparent that students needed to have core clinical competencies and skills allied to their own profession in order to gain maximum benefit from the various learning opportunities offered by the workshop. Medical students in particular need to have basic mathematical skills, be aware of the various sources of information and able to accurately complete a Drug Kardex. These issues may need addressed at earlier stages in the curriculum.

Despite the resource implications, timetabling considerations and time and commitment required from educators to allow these workshops to be delivered, the positive evaluation supports the expansion of the workshops to other student groups and to include other prescribing areas. In other medical schools, 4^th ^year paediatric students and final year pharmacy students jointly participated in a paediatric workshop^12 ^suggesting that this workshop is appropriate for other disciplines and has potential to be extended to other areas of clinical practice and to other paediatric medications.

## Conclusion

This study has demonstrated that shared learning is an effective approach to achieving common learning outcomes for undergraduate medical and nursing students. Participation in the workshop has helped bridge the knowledge-skills gap and provided an opportunity for students to develop and enhance their communication and teamworking skills.

An interprofessional teaching and learning approach to safe paediatric drug prescribing and administration has the potential to improve quality and safety within healthcare. However, long term follow up is needed to determine if healthcare students are more competent practitioners following their participation in this study.

### Study limitations

#### Short term follow up

It is unclear whether learning outcomes translate into improved safety in workplace.

IPE student groups limited to medical and nursing students.

### Study implications

Study addresses current safety concerns re drug prescribing and administration.

IPE approach facilitates communication and teamworking in addition to knowledge and skills.

Practical learning experience relevant to future practice.

Qualitative and quantitative evidence of benefits of IPE approach to learning, drug prescribing and administration.

## Competing interests

The authors declare that they have no competing interests.

## Authors' contributions

MCS: Planned, implemented and evaluated workshops. Principal author. JP: Co-ordination of workshops, major contribution to preparation of paper. AB: Planned and implemented workshop, expert advice on pharmacology of paediatric drug prescribing and administration. NK: Planned, implemented and evaluated workshops and advised on manuscript preparation. All authors read and approved the final manuscript.

## Pre-publication history

The pre-publication history for this paper can be accessed here:

http://www.biomedcentral.com/1472-6920/10/19/prepub
